# Supplementary material for: Assessment of the Activity of Decoquinate and Its Quinoline-O-Carbamate Derivatives against Toxoplasma gondii In Vitro and in Pregnant Mice Infected with T. gondii Oocysts
Source: Molecules. 2021 Oct 22;26(21):6393. doi: 10.3390/molecules26216393 (PMC8587999; doi:10.3390/molecules26216393)
Supplement: Supplementary file 1 [file molecules-26-06393-s001.zip › molecules-1401068-supplementary.pdf]

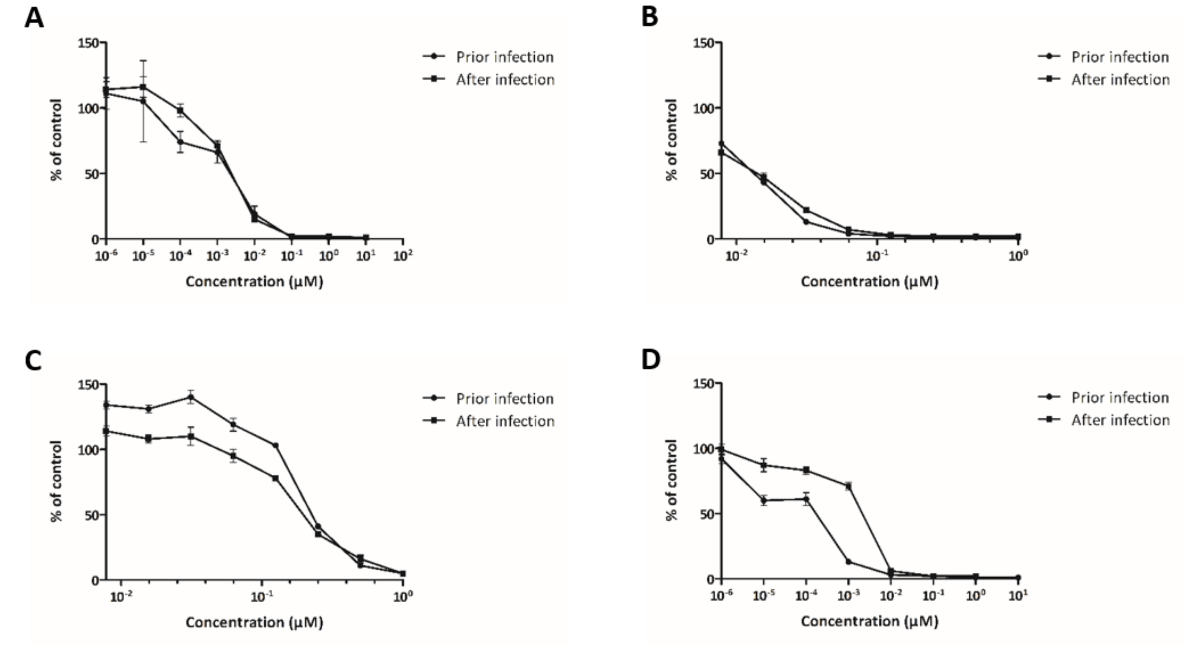

Figure S1. IC50 dose-response curves for *T. gondii* tachyzoites grown in HFF treated with DCQ (A), RMB054 (B), RMB055 (C) and RMB060 (D). Compounds were either added prior or 3h after infection with Tg-β-gal.

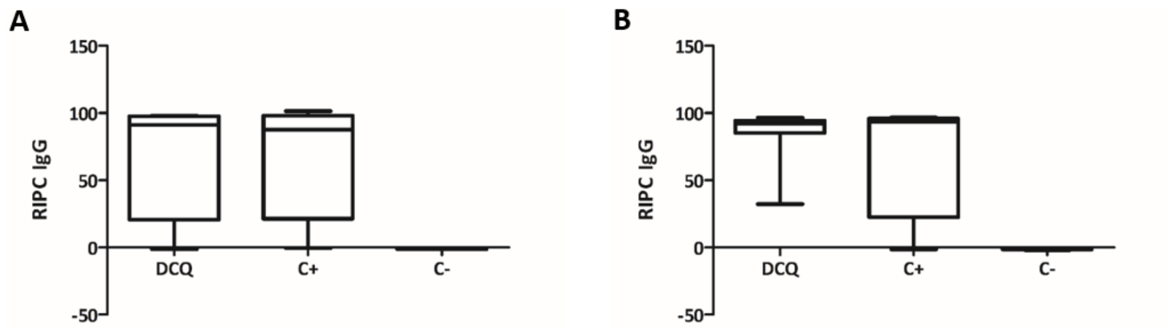

Figure S2. *T. gondii* IgG antibody titers of non-pregnant mice (A) and dams (B) measured from serum collected at the end of the experiment. Results are depicted as the mean of RIPC (relative index per cent) compared to the positive control (C+). In non-pregnant mice and dams, no statistically significant differences in the IgG antibody titers were observed between DCQ-treated mice compared to the C+ group.

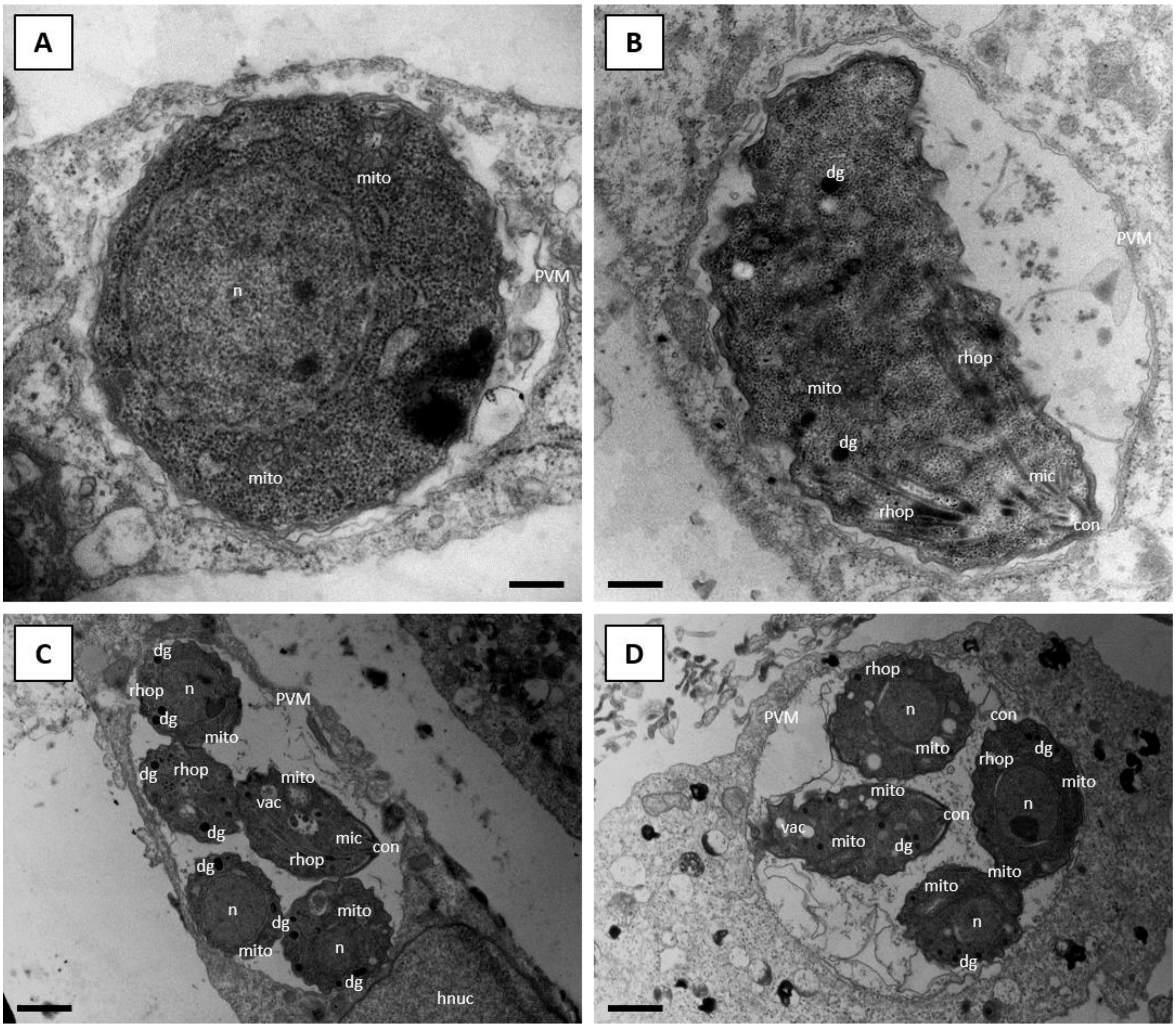

Figure S3. TEM of *T. gondii* Me49 tachyzoites cultured *in vitro* in HFF host cells. HFF monolayers were infected with *T. gondii* tachyzoites and drug treatment was initiated 3h later. TgMe49 treated with 0.5 µM DCQ for 6h are shown in (A), 24h in (B), 48h in (C) and 72h in (D). con = conoid; dg = dense granules; mic = micronemes; mito = mitochondrion; n = nucleus; hnuc = host cell nucleus; PVM = parasitophorous vacuole membrane; rhop = rhoptries; vac = cytoplasmic vacuole. Bars in (A) = 0.4 µm; (B) = 0.4 µm; (C) = 2.2 µm; (D) = 1.8 µm.

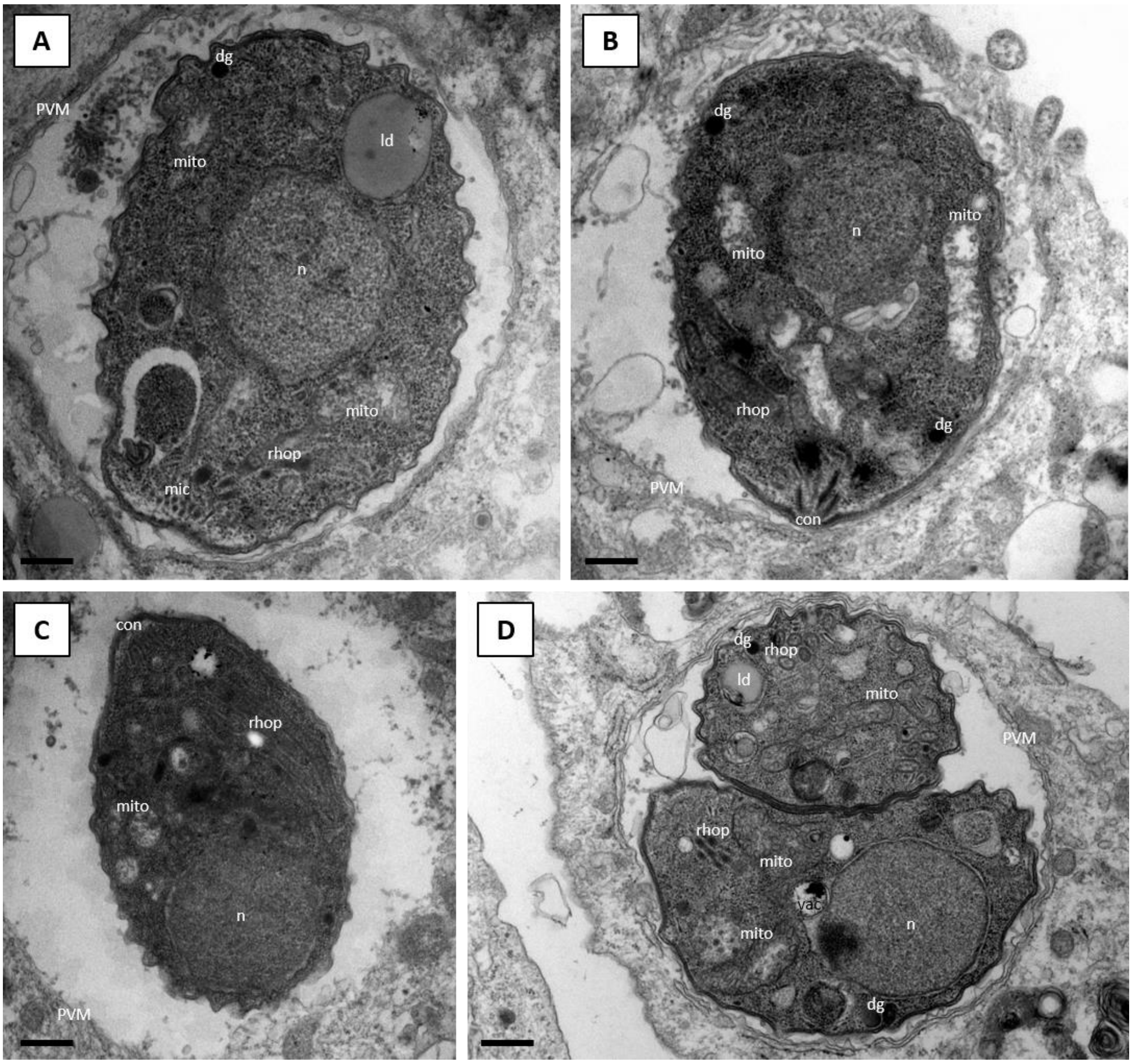

Figure S4. TEM of *T. gondii* Me49 tachyzoites cultured *in vitro* in HFF host cells. HFF monolayers were infected with *T. gondii* tachyzoites and drug treatment was initiated 3h later. TgMe49 treated with 0.5 µM RMB054 for 6h are shown in (A), 24h in (B), 48h in (C) and 72h in (D). con = conoid; dg = dense granules; ld = lipid droplet; mic = micronemes; mito = mitochondrion; n = nucleus; PVM = parasitophorous vacuole membrane; rhop = rhoptries; vac = cytoplasmic vacuole. Bars in (A) = 0.4 µm; (B) = 0.4 µm; (C) = 0.6 µm; (D) = 0.6 µm.

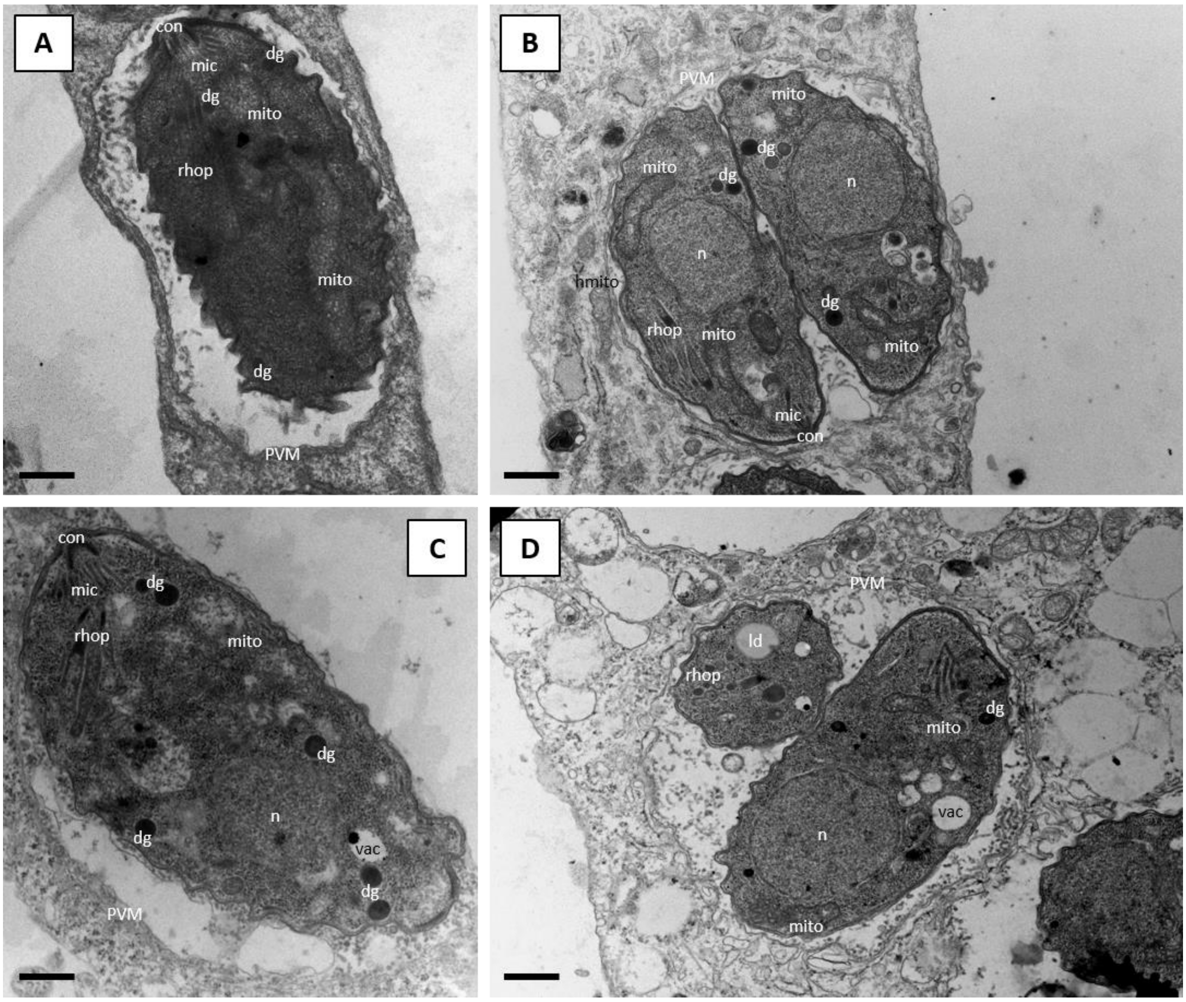

Figure S5. TEM of *T. gondii* Me49 tachyzoites cultured *in vitro* in HFF host cells. HFF monolayers were infected with *T. gondii* tachyzoites and drug treatment was initiated 3h later. TgMe49 treated with 1 µM RMB055 for 6h are shown in (A), 24h in (B), 48h in (C) and 72h in (D). con = conoid; dg = dense granules; ld = lipid droplet; mic = micronemes; mito = mitochondrion; hmito = host cell mitochondrion; n = nucleus; PVM = parasitophorous vacuole membrane; rhop = rhoptries; vac = cytoplasmic vacuole. Bars in (A) = 0.6 µm; (B) = 1 µm; (C) = 0.6 µm; (D) = 1 µm.

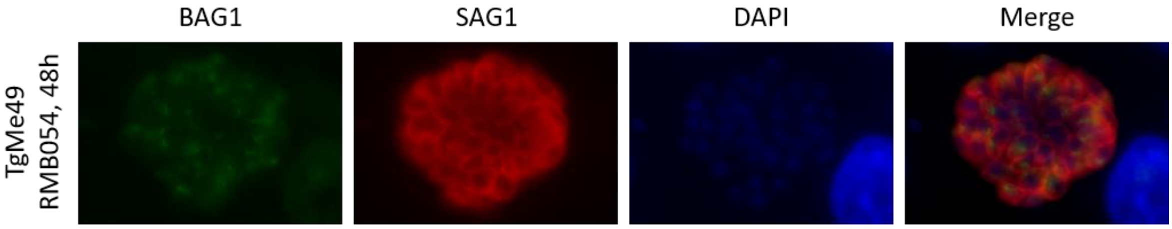

Figure S6. Immunofluorescence staining of HFF monolayers infected with *T. gondii* Me49 (TgMe49) tachyzoites after treatment with RMB054 for 48h.

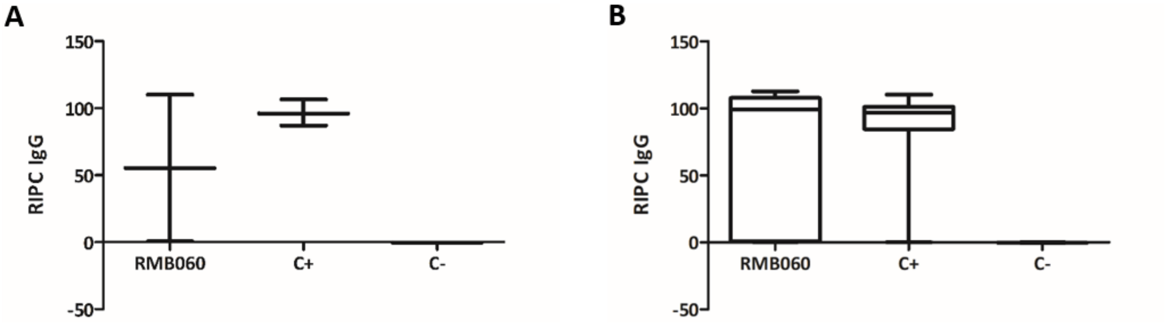

Figure S7. *T. gondii* IgG antibody titers of non-pregnant mice (A) and dams (B) measured from serum collected at the end of the experiment. Results are depicted as the mean of RIPC (relative index per cent) compared to the positive control (C+). In non-pregnant mice and dams, no statistically significant differences in the IgG antibody titers were observed between RMB060-treated mice compared to the C+ group.
